# Frequency of Alu insertions within the *ACE* and *PR* loci in Northwestern Mexicans

**DOI:** 10.1186/s13104-017-2673-y

**Published:** 2017-07-27

**Authors:** Hilda P. Navarrete, Linda H. Soler, Rosa E. Mares, Marco A. Ramos

**Affiliations:** Facultad de Ciencias Químicas e Ingeniería, Universidad Autónoma de Baja California, Calzada Universidad 14418, Parque Industrial Internacional, 22390 Tijuana, BCN Mexico

**Keywords:** Human polymorphisms, Alu insertions, Genotyping, Mexican population, Genomic diversity

## Abstract

**Objective:**

Presently, non-LTR retrotransposons are the most active mobile elements in the human genome. Among these, Alu elements are highly represented in the modern population. Worldwide, distribution of Alu polymorphisms (insertion/deletion; I/D) shows variability between different populations. Two Alu insertion loci, *ACE* and *PR*, are significant biomarkers that have served in several genotype–phenotype association studies. In Mexico, studies concerning the frequency of these biomarkers have been conducted mainly in subpopulations from central and southern regions. Here, we screened a population sample of the northwestern region to gain further knowledge regarding the prevalence of Alu polymorphisms within *ACE* and *PR* loci.

**Results:**

For *ACE* locus, the observed genotype frequencies were 26.5, 51.0 and 22.5% for II, ID, and DD, respectively; and allelic frequencies for I and D were 52 and 48%. Whereas respective genotype frequencies for *PR* locus were 2.7, 26.5 and 70.8%, and the corresponding allele frequencies were 16 and 84%. Furthermore, the insertion frequency within *ACE* locus was similar between central, western and northwestern subpopulations, and rather higher in southeastern subpopulation (*p* < 0.05). Although the occurrence of Alu polymorphisms within *PR* locus has not been widely examined, the insertion frequency was higher in northwestern subpopulation, as compared with western and southeastern subpopulations (*p* < 0.05). Based on the frequency of Alu insertions found in *ACE* and *PR* loci, subpopulations from the northwestern, western and central regions share a common genetic origin, but apparently not with the subpopulation from the southeastern region, in accordance with the notion that assumes the existence of a broad genomic diversity in the Mexican population. In addition, the high prevalence of Alu insertions reveals their potential application as biomarkers with prognostic value for the associated diseases; e.g., as part of the standard protocols for clinical diagnosis.

## Introduction

Mobile elements can be classified as either DNA transposons or retrotransposons. DNA transposons are currently not mobilizing in the human genome, while retrotransposons have significant mobility. Retrotransposons can be subdivided into LTR and non-LTR elements, which are distinguished by the presence or absence of long terminal repeats. LTR retrotransposons are endogenous retroviruses with very limited activity. By contrast, non-LTR retrotransposons, typified by L1, Alu, and SVA elements, are presently the most active [1–3]. Therefore, numerous de novo insertions have resulted in human diseases [3–6].

Alu elements are one of the most represented in the human genome, being about one million copies (almost 11% of the genome). As active elements in the modern population, new insertions into somatic cells contribute to genomic diversity, gene mutations, and genetic diseases [7, 8]. In addition, the ubiquitous presence of Alu insertions has culminated in their appearance in many genes and transcripts, having a far-reaching influence on gene expression [7, 9, 10]. Moreover, it has been suggested that the most detrimental effect is the interaction between highly homologous elements and their potential to generate deletions, duplications, inversions and other complex genomic rearrangements [11]. Overall, about 0.5% of all human genetic disorders, including some types of cancer, have allegedly resulted from Alu-mediated unequal homologous recombination [12].

The insertion/deletion (I/D) polymorphism of the angiotensin-converting enzyme (*ACE*) locus is an important genetic biomarker that has served in numerous genotype–phenotype association studies [13]. *ACE* plays a key role in the regulation of systemic blood pressure and renal electrolyte homeostasis by converting the inactive angiotensin I into the potent vasoconstrictor and aldosterone-stimulator angiotensin II, and by inactivating the pro-inflammatory vasodilator bradykinin [13, 14]. The I/D polymorphism is distinguished by the presence (insertion) or absence (deletion) of an Alu element within intron 16 of the *ACE* locus, resulting in three different genotypes: II, ID and DD. Moreover, it seems that the serum concentrations of *ACE* correlate with the I/D polymorphism (DD > ID > II), suggesting that the levels of circulating enzyme may be determined by the genotype at the *ACE* locus [15].

The long-range haplotype, named PROGINS, found in the progesterone receptor (*PR*) locus comprises an Alu insertion within the intron G (between exon 7 and 8) and two single-nucleotide polymorphisms (SNP): G→T transversion in exon 4, producing a missense mutation (V660L), and C→T transition in exon 5, yielding a silent mutation (H770H). Remarkably, the identification of any of these three alleles uniquely recognizes the presence of the other two [16]. The phenotypic effect of PROGINS is predicted to be due to both the Alu insertion, affecting gene expression and RNA stability, and the V660L substitution, leading to a reduction in the response to progesterone [17].

Distribution of Alu polymorphisms shows variability among different world populations [18]. In Mexico, studies concerning the frequency of these biomarkers have been conducted mainly in populations of central and southern regions [19–21]. Thus, to obtain additional data on the distribution of Alu insertions among other subpopulations, we screened a sample of the northwestern region. Here, we report the prevalence of Alu polymorphisms within *ACE* and *PR* loci, two genomic variations which presumably can lead to diseases in humans. Moreover, these biomarkers offer the possibility of being translated to clinical practice after a thorough validation of the genotype–phenotype association.

## Main text

### Methods

One hundred and forty-seven samples were available from a DNA biobank collected in a previous study [22]. Any personal data was removed from the sample tube prior to conduct this study; e.g., ensuring individual privacy and autonomy [23]. All samples were assayed for each Alu polymorphism; e.g., *ACE* or *PR*. Each segment comprising the insertion was amplified by polymerase chain reactions (PCR) using locus-specific primers (Table 1). Although these assays are sensitive and well documented, precision and reproducibility were ensured by re-typing randomly selected samples throughout the study.

**Table 1:** The sequence of locus-specific primers and length of PCR products used for discrimination of Alu polymorphisms

Locus	Primer sequence (5′–3′)	PCR product (bp)
*ACE*	FW: CTGGAGACCACTCCCATCCTTTCT	Insertion (I): 480
	RV: GATGTGGCCATCACATTCGTCAGAT	Deletion (D): 191
*PR*	FW: GGCAGAAAGCAAAATAAAAAGA	Insertion (I): 479
	RV: AAAGTATTTTCTTGCTAAATGTC	Deletion (D): 159

Typical PCR amplifications were performed in a total volume of 0.02 mL of 1X Taq Mix (Qiagen) containing 25 pmol of each primer and 100 ± 40 ng of template DNA. Thermal cycling conditions were as follows: an initial denaturation step (2 min at 94 °C), followed by 35 cycles of exponential amplification (20 s at 94 °C, 20 s at 50 °C, and 40 s at 72 °C), and a final elongation step (7 min at 72 °C). PCR products were separated by gel electrophoresis (2% agarose) and stained with ethidium bromide. The I/D polymorphisms were determined by visual discrimination of each fragment length (Table 1).

The observed values for allele and genotype frequencies were obtained by direct counting, while the expected value for genotype frequency was calculated according to the Hardy–Weinberg (HW) model. HW equilibrium was verified by the goodness-of-fit test χ^2^ with a significant confidence value of *p* < 0.05.

### Results

Since a significant percentage (around 0.5%) of all human genetic disorders might result from Alu-mediated unequal homologous recombination [12], the discovery and characterization of the insertion sites are essential in narrowing down the cause of such diseases [24]. Moreover, as Alu elements are the most abundant interspersed repeats in the human genome [8], the distribution and classification of widespread variants are decisive to identify novel pathogenic insertions [24, 25].

We have reported the prevalence of three pharmacogenetic traits in northwestern Mexicans [22]. To gain additional data regarding other biomarkers, a genetic screening was performed to determine the frequency of Alu polymorphisms within *ACE* and *PR* loci (Table 2). For *ACE* locus, the observed genotype frequencies for II, ID and DD were 26.5, 51.0 and 22.5%; and the frequencies for I and D alleles were 52 and 48%; respectively. Whereas for *PR* locus, the observed frequencies for II, ID and DD genotypes were 2.7, 26.5 and 70.8%; and the allele frequencies were 16 and 84% for I and D; respectively. Furthermore, distribution of polymorphic genotypes was found to be in Hardy–Weinberg equilibrium (*p* > 0.05).

**Table 2:** The frequencies of the Alu polymorphisms found within *ACE* or *PR* locus among northwestern Mexicans (*N* = 147)

Locus	Genotype (*f*)	Allele (*f*)	*P* value (χ^2^)
*ACE*	II: 39 (0.265) ID: 75 (0.510) DD: 33 (0.225)	I: 153 (0.52) D: 141 (0.48)	0.789 (0.072)
*PR*	II: 4 (0.027) ID: 39 (0.265) DD: 104 (0.708)	I: 47 (0.16) D: 247 (0.84)	0.881 (0.022)

Through a comparative analysis (Table 3), we found that the frequency of Alu insertions within *ACE* locus was quite similar between central, western and northwestern subpopulations, but significantly different in a southeastern subpopulation (*p* < 0.05), suggesting distinct genetic origins amongst current Mexican subpopulations. Conversely, although the occurrence of Alu polymorphisms within *PR* locus has not been widely examined, we found that the frequency of insertions was significantly higher in a northwestern subpopulation, as compared with western and southeastern subpopulations (*p* < 0.05), supporting the previously mentioned notion.

**Table 3:** The frequency of the Alu insertions within *ACE* or *PR* locus among Mexican subpopulations

Locus	Subpopulation (region)	*N*	*F* (I)	References
*ACE*	Baja California (NW)	147	0.520	This study
	Jalisco (W)	144	0.517	[35]
		288	0.519	[36]
	Michoacán (W)	269	0.572	[37]
	México D.F. (C)	138	0.590	[38]
		98	0.602	[19]
	Yucatán (SE)	51	0.735	[39]
*PR*	Baja California (NW)	147	0.160	This study
	Jalisco (W)	209	0.079	[21]
	Campeche (SE)	48	0.060	[20]

### Discussion

Because of their transposition activity, Alu elements represent a significant source of genomic variation [2]. Their importance becomes highlighted by the potential association with genetic instability, one of the major causes of human diseases such as cancer [26]. In addition, several studies have demonstrated their ability to modulate gene expression at the post-transcriptional level [27]. From the evolutionary standpoint, the Alu elements can be regarded as fixed or polymorphic. Fixed elements are evolutionarily older and present throughout the population, while those that are polymorphic are the result of recent retrotransposition events and can be found in a subset of individuals in the population. Since the prevalence of polymorphic alleles may vary between populations of different origin [28], genotype screening is essential identifying individuals carrying insertions (e.g., homozygous and heterozygous), especially when the genotype–phenotype association has been well established, because those individuals could be at risk of developing the condition associated [7, 24, 29, 30].

In Mexican populations, the ancestral genetic contribution exhibits regional fluctuations [31]. Although numerous studies have been conducted in different regions, a few have been performed in the northwestern region. Since polymorphic Alu elements are reliable biomarkers [32, 33], we used those within *ACE* and *PR* loci to determine the occurrence of I/D polymorphism in the northwestern subpopulation. Our results showed that the frequency of Alu insertions found in the examined subpopulation is quite comparable to the observed in western and central subpopulations, but it is significantly different from that reported in southeastern subpopulation (*p* < 0.05). This is consistent with the notion that presumes the existence of a broad genomic diversity in the Mexican population [31, 34].

## Limitations

The high prevalence of Alu insertions in current Mexican population reveals their potential application as biomarkers with a prognostic value for the associated diseases; e.g., as part of the standard protocols for laboratory diagnosis. However, this notion should be properly assessed through genotype–phenotype association studies to adequately fulfill clinical purposes.

## Abbreviations

LTR: long terminal repeats
*ACE*: angiotensin converting enzyme
*PR*: progesterone receptor
SNP: single nucleotide polymorphisms
